# Indirect evaluation of pit and fissure sealants: A 3D-based method validation

**DOI:** 10.4317/jced.56688

**Published:** 2020-09-01

**Authors:** Kelly Moreira, Kamila Kantovitz, Tamires Bueno, Maria-Angélica Agulhari, Fabio Rizzante, Juliana Aguiar, Fernanda Pascon, Vanessa Arias, Ana-Flávia Borges, Regina-Maria Rontani

**Affiliations:** 1 Department of Operative Dentistry, Endodontics and Dental Materials. Bauru School of Dentistry-FOB-USP

## Abstract

**Background:**

The aim of the present study was to compare indirect methods to assess the clinical performance of pit and fissure sealants and validate the use of 3D scanners.

**Material and Methods:**

Sample consisted of 58 plaster models of upper and lower first permanent molars, sealed with resin sealants, as well as photographs obtained during the 18-month follow-up. Pre-established criteria were applied to categorize the sealant presence/absence and marginal integrity. Two calibrated examiners performed the evaluations, independently, using Scanning Electron Microscopy (SEM; gold-standard), Photography, 3D (CEREC In Lab) and Stereomicroscope analysis.

**Results:**

The intra-examiner Spearman correlation was 94% e 97%, respectively, and the inter-examiner was 96%. Data was submitted to Kappa test, Spearman correlation and Receiver Operating Characteristic Curve (ROC). 3D and SEM presented good concordance; Stereomicroscope showed regular concordance with SEM and 3D (*p*<0.001). There was no concordance among Photography and the other methods (*p*>0.05). SEM had a significant positive correlation with 3D and Stereomicroscope (r=0.76 and 0.71, respectively; *p*<0.01). There was significant positive correlation (r=0.65) between 3D and Stereomicroscope (*p*<0.01). The ROC estimated curve areas for Stereomicroscope and 3D were 0.90 (IC:0.81-0.99) and 1.0 (IC:1.0-1.0), respectively (*p*<0.001).

**Conclusions:**

Photography presented lower sensitivity and specificity (area=0.59). 3D method showed the best performance when compared to gold standard, exhibiting high sensitivity and specificity, therefore, it was validated as a reliable method to evaluate pit and fissure sealants.

** Key words:**Photography, stereomicroscope, SEM, diagnostic, sealants.

## Introduction

Fissure sealants are one of the most effective approaches to prevent or arrest caries lesion development on occlusal surfaces in patients with high-caries-risk (1-6). This strategy aims to establish a long-term protective physical barrier, restraining acid-producing microorganisms from access to their source of nutrients in pits and fissures (2,4,6,7). However, sealants deteriorate over time. This process often results in displacement of small or large parts of material, re-exposing the enamel surface to oral environment and, consequently, increasing risk of carious lesions development in some patients (2,4,7).

Sealant retention should be carefully analyzed since it has as goal caries prevention (4-7). Literature reports retention playing a major role in the maintenance of sound pits and fissures, especially when a resin-based sealant is used (6-10). Considering resin-based materials, the first sign of failure is the presence of marginal gap leading to marginal staining and generating interfacial stresses(11), which can result in sealant de-bonding from tooth structure (10,12) .

Evaluation of dental sealants integrity through traditional methods, such as visual and probing inspection, have been found to provide far from ideal feedback (13). Despite being conducted by trained and calibrated evaluators using artificial light, mirrors and probes, clinical examination has few shortcomings. The most remarkable is the inability to recall/reassess a previously made observation (14). Other limitations include the relatively short time in which sealants are assessed, light reflection by some materials hindering proper visibility and examiner fatigue over time. All those shortcomings may result in unreliable observations (8,15). Indirect methods such as standardized color photographs, stereomicroscope, and scanning electron microscopy, have been suggested as well-established strategies to overcome such limitations (8,15). Such methods have been applied to detect developmental enamel defects (16) and to measure the cement thickness in crowns(17). Photographic method has been used for caries lesion diagnosis and materials performance evaluation on the occlusal surface (18-20), while stereomicroscopes are used in surgeries, as well as studies evaluating occlusal surface (21).

Digital dentistry is an emerging field, and the acquisition of virtual 3D models through intra/extra oral scanners could optimize dental office workflow (17). Digital tools create a completely new range of possibilities for new investigations that go beyond prosthetic restorations. The evolution of these systems associated with evolution in dental material results in more predictability and preservation of sound tooth structure. Replica and virtual methods may be more sensitive than visual clinical examination in measuring the levels of retention and degradation of occlusal sealants overtime (22). The main advantages of using 3D methods is the permanent availability of the 3D models, the use of convenient and commonly available tools (such as intraoral scanners or regular benchtop scanners), possibility of image segmentations and specific analyses, among others (16). These methods are thought to reduce operator fatigue and consequently, evaluation bias (16). However, in the best of author’s knowledge, there is no study comparing the reliability of the different indirect evaluation methods: bidimensional (SEM, Stereomicroscope and Photographs) and tridimensional (3D models), to assess marginal integrity and resin sealants retention.

Therefore, the aim of the current investigation was to compare indirect methods to assess the clinical performance of pit and fissure sealants and to validate the evaluation using a 3D model system. The null hypothesis was that there would be no significant differences among the methods (Photographic, Stereomicroscope, Scanning Electronic Microscope and 3D) in assessing the marginal integrity and resin sealants retention.

## Material and Methods

-Experimental Design 

This study had as study factor the different evaluation methods in 4 levels (SEM, Stereomicroscope, 3D and Photographs). Experimental units were composed by first permanent molars having as response factors marginal integrity and retention of resin pits and fissures sealants.

After approved by the Local Ethics Committee (protocol #143/2003), data of children aged 6-10 years, who received sealants in their permanent first molars were evaluated (n=58). Data included stone models and photographs obtained during the 18-months follow up session.

-Stone cast models

Type IV stone casts (Durone, Dentsply Industria e Comércio Ltda., RJ, Brazil) were stored at 37ºC for dehydration, gold-sputtered (Bal-Tec SCD 050 Sputter Coater, Bal-Tec; Balzers, Liechtenstein) and analyzed with SEM (JEOL- JSM 5600LV, Tokyo, Japan) at 15 kV, with 20mm working distance and 100X magnification.

-Photographs

A Sony Mavica MVC-FD97 (SonyLens/Optical 10x f=6.0-60.0mm 1:2.8 Ø52, 2.1 megapixels CCD, Sony, Tokyo, Japan) was used to take the pictures during the follow up session without zoom. Files were stored as JPEG file.

-3D (3D Models)

Stone cast models were digitized using a benchtop 3D scanner (Scanner InEos Blue, Sirona, Bensheim, Germany) with Cerec Inlab SW4 software (Sirona), 486x584 pixels resolution. All the images were converted to JPEG for evaluation.

-Stereomicroscope 

A stereomicroscope (LEICA- MZ6, Wetzlar, Germany) was used at 20X magnification to examine the stone model surfaces. The casts were adapted in the stereomicroscope using wax.

Each sample was evaluated eight times (two for each method, with one-week interval) by each of two calibrated investigators.

-Statistical analysis

Data was submitted to Kappa coefficient test (κ), Spearman correlation and Receiver Operating Characteristic Curve (ROC), adopting 5% significance level. Data was organized into excel spreadsheet and analyzed using SPSS 21 (IBM®, Chicago, USA) software.

For ROC, authors tested a null hypothesis in which the area below the curve would be equal to 0.5, versus the alternative hypothesis in which the area would be bigger than 0.5.

## Results

G-Power program was used to calculate power test considering a sample of 56 and the power of the test was 0.84. Calibration was performed through a preliminary pilot study in order to access the reproducibility of the evaluation criteria (Table 1). Consistency among the results for each method was assessed through two-way Kappa’s test. Kappa’s results revealed lower concordance between photographic and SEM (κ=0.065, *p*=0.43), and stereomicroscope (κ=0.089 *p*=0,319), as well as lack of concordance between photographic and 3D methods (κ=0.021, *p*=0.791). The 3D method presented substantial concordance with SEM (κ= 0.685, *p*<0.001) and with stereomicroscope (κ=0.40, *p*<0.001). Stereomicroscope and SEM methods also showed fair agreement (κ=0.354, *p*<0.001).

In addition, Spearman’s correlation test was applied to verify a possible association among the 4 methods (Table 2). Photographic evaluation did not show correlation with any of the other tested methods (SEM – 0.251, 3D model – 0.217, or Stereomicroscope – 0.126). SEM showed a strong correlation with 3D model (0.757) and Stereomicroscope (0.714). 3D model and Stereomicroscope also showed good correlation (0.650).

**Table 1:**
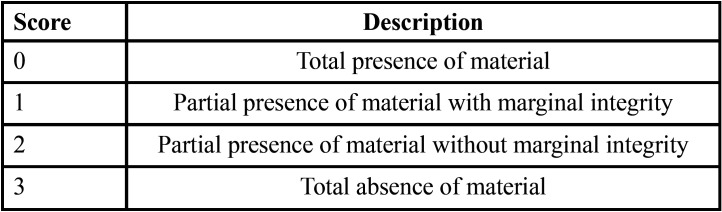
Criteria used for the assessment sealants (adapted from Feigal *et al.*, 2000) (24).

**Table 2:**
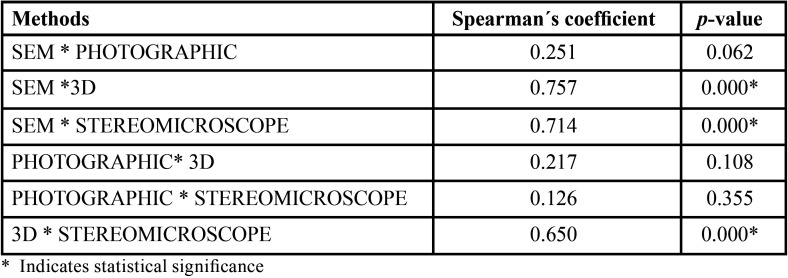
Spearman´s coefficient between different diagnosis methods.

**Figure 1:**
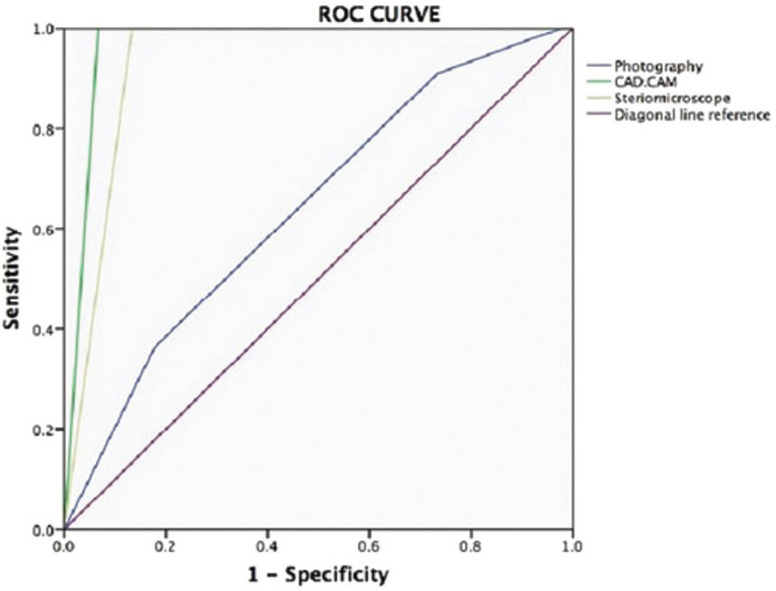
Receiver Operating Characteristic Curve to indirect methods in relation to SEM.

In addition, the measurements of sensitivity and specificity among the different methods were estimated and the acquired ROC curve can be observed in Figure 1. ROC curve generated areas bigger than 0.5 for stereomicroscope (0.898), 3D method (1,0), and photographic method (0.585). ROC curve evidenced lower sensitivity and specificity for photographic method generating an area of 0.585.

## Discussion

Based on the results of the present study, the null hypothesis was rejected (*p*<0.001), and proved that both methods have a good sensitivity and specificity when compared with gold stand parameter (SEM).

Different diagnostic methods have been proposed to assess carious lesions and direct/indirect restorations, as well as evaluation of fractures, marginal infiltration and retention of different restorative materials. Although direct visual method is often used to identify the integrity of sealants (23), presence of saliva, poor illumination, and a short time period turns it into a challenging procedure. This said, the use of indirect methods, especially digital tools, could improve the preventive, restorative and rehabilitator research/clinical workflow.

Sensitivity and specificity tests are used to compare new diagnostic methods with a chosen gold standard. The sensitivity is important to determine the probability of positive diagnostic when the result should be positive; and the specificity determine the probability of negative outcome when the results should be negative. A new method can be considered adequate when it shows similar-to-higher sensitivity and specificity values when compared with a gold standard(23). In the present study, SEM was considered as the gold standard due to its high sensitivity and specificity (8).

Some studies suggested the use of photographs as a more reliable method for caries assessment and sealant retention scoring when compared with clinical analysis, especially due to possibility of magnification and lack of time limitations (18,23). Nevertheless, the present study results showed a lower sensitivity and specificity when compared with other methods regarding assessment of sealant retention, not allowing a proper diagnosis. Similar to other reports, the results could have been jeopardized due to the difficulties in standardizing and getting clean and focused photos (19,20).

Stereomicroscope can be used for clinical as well as for research purposes, being suiTable for microleakage assessment of pit and fissure sealants (12,21), showing high sensitivity and specificity in the present study.

With the recent development of digital workflow in dentistry, the use of 3D systems (i.e. intra and extra oral scanners) to create digital models is becoming very popular, and with the improvements in their accuracy, might be suiTable as a research tool (17). The results of the present study corroborate it, since 3D method showed a strong correlation with the SEM method (0.757) and presented great sensitivity and specificity (1.0). This said, the use of 3D models can be considered a valuable tool to assess retention and marginal integrity of sealants and shows advantages such as the possibility of rotating and zooming the samples, allowing assessment from several different perspectives. It is noteworthy that an adequate stone cast is mandatory in order to allow proper assessment when using some extra-oral scanners, although some recent scanners are able to generate 3D models based on impressions. Another possibility also consists in the use of intraoral scanners, which is becoming more frequently available in dental offices, dental schools and research centers. Such approaches might be able to reduce costs, time, patient discomfort, as well as improve the accuracy since it could minimize human errors and inherent materials’ distortion. In addition, some companies allow the 3D acquired data to be exported to other file formats, increasing the number of softwares, and consequently, methods, that could be used to study different parameters and/or procedures, projecting 3D based methods as an even more versatile and powerful research tool.

Another interesting use of 3D models can consist in a self-assessment and quality control of procedures performed by dentists in their offices in a daily basis, especially because current softwares can superimpose different models overtime, allowing comparison of different restoration’s properties such as retention, anatomy, wear, among others.

Future studies assessing long term wear rate and retention of sealants and/or restorative materials should be performed using digital technologies, superimposing the digital models from different timeframes and allowing a precise assessment of the alterations.

In conclusion, 3D method showed better specificity and sensitivity to evaluate the loss and conditions of occlusal sealants compared to SEM (gold standard). Therefore, this indirect method can be used to assess the clinical performance of pit and fissure sealants.
